# Helicobacter pylori Infection and Complications of Cirrhosis

**DOI:** 10.7759/cureus.54419

**Published:** 2024-02-18

**Authors:** Shefali Amin, Biraj Shrestha, Ameya Deshmukh, Manish Shrestha, Parth Desai, John Altomare

**Affiliations:** 1 Internal Medicine, Tower Health Medical Group, Reading, USA; 2 Cardiovascular Medicine, Lehigh Valley Health Network, Allentown, USA; 3 Internal Medicine, Saint Louis University School of Medicine, St. Louis, USA; 4 Gastroenterology and Hepatology, Tower Health Medical Group, Reading, USA

**Keywords:** hepatorenal syndrome, helicobacter pylori, gastrointestinal (gi) bleeds, hepatic encephalopathy, cirrhosis, h. pylori infection

## Abstract

Introduction: *Helicobacter pylori* is a significant contributor to conditions such as peptic ulcer disease, gastric cancer, gastric mucosa-associated lymphoid tissue lymphoma, and colorectal cancer. Recent studies have suggested a potential link between *H. pylori* and cirrhosis. However, the impact of *H. pylori* on cirrhosis-related mortality, inpatient outcomes, and decompensating events remains unclear. Considering the widespread availability of *H. pylori* testing and effective treatment options, there is a potential rationale for eradicating *H. pylori* in cirrhotic patients to mitigate the morbidity and mortality associated with cirrhosis. This study aims to investigate the association between *H. pylori* and inpatient outcomes and complications related to cirrhosis.

Methods: The National Inpatient Sample (NIS) database, a part of the Healthcare Cost & Utilization Project, was utilized for this study. Inpatient data from the years 2016 through 2019 were extracted for patients with a primary discharge diagnosis of cirrhosis and a concurrent diagnosis of *H. pylori* infection. The primary outcomes included inpatient mortality, length of stay, and cost of care. Secondary outcomes involved cirrhosis-related complications during hospitalization, such as gastrointestinal bleeding, hepatic encephalopathy, and hepatorenal syndrome.

Results: Over the years 2016 to 2019, 416,410 patients received a primary discharge diagnosis of cirrhosis. Among them, 990 patients (0.2%) had a secondary diagnosis of *H. pylori* infection. Those with both cirrhosis and *H. pylori* tended to be younger on average (mean age 54.25 vs. 57.18 years, p=0.01) and more frequently fell within the age range of 18-49 (33.84% vs. 24.71%, P=0.01). *H. pylori*-infected patients were also more likely to be male (70.71% vs. 63.11%, P<0.028), of Hispanic race (36.4% vs. 18.6%, p< 0.1), and of Black race (20.2% vs. 8.1%, p< 0.1). While *H. pylori*-exposed patients had lower in-hospital mortality (0.51% vs. 4.44%, p=0.007), their mean length of stay was higher (6.97 days vs. 5.75, p=0.002). The overall cost of care was comparable between the *H. pylori*-exposed and non-exposed groups (mean USD18,106.18 vs. $16,543.49, P=0.160). *H. pylori*-exposed patients had a higher overall rate of cirrhosis-related complications (84.85% vs. 67.59%, p< 0.001), gastrointestinal bleeding (48.48% vs. 27.34%, p< 0.001), and hepatorenal syndrome (70.71% vs. 46.99%, p< 0.001), and these differences persisted in multivariable analysis. Initially, rates of hepatic encephalopathy were higher in *H. pylori* non-exposed patients (21.57% vs. 15.66%, p=0.04), but this discrepancy was corrected after adjusting for potential confounders.

Conclusion: While patients in this study were diagnosed with both *H. pylori* and cirrhosis by discharge, it cannot be definitively concluded that *H. pylori* was the direct cause of cirrhosis complications. Recognizing this uncertainty, further studies are needed better to understand the associations between cirrhosis and *H. pylori* complications. Distinguishing the causes of cirrhosis and its relationship with *H. pylori* may offer deeper insights into whether *H. pylori* is a causative factor or merely correlated in its effects on patients with cirrhosis.

## Introduction

*Helicobacter pylori* is the most common chronic infection worldwide, affecting over 50% of the global population [1]. It is a major cause of peptic ulcer disease, gastric cancer, gastric mucosa-associated lymphoid tissue lymphoma, and colorectal cancer [2-3]. In addition, as *H. pylori* may induce a chronic inflammatory state, it has been linked to extraintestinal manifestations such as immune thrombocytopenic purpura, coronary artery disease, iron deficiency anemia, and migraines [4].

In recent years, clinicians have been investigating a potential link between *H. pylori* and cirrhosis. In one meta-analysis, patients with cirrhosis were significantly more likely to be infected with *H. pylori* when compared to controls [5]. However, the contribution of *H. pylori* to cirrhosis mortality, inpatient outcomes, and decompensating events remains unclear. Given the widespread availability of *H. pylori* testing and effective treatment strategies, eradicating *H. pylori* in cirrhotic patients may be warranted to reduce morbidity and mortality associated with cirrhosis if a link exists. The present research focuses on exploring the association between *H. pylori* and cirrhosis-related inpatient consequences and complications.

This article was previously presented as a meeting abstract at the 2022 American College of Gastroenterology (ACG) Annual Scientific Meeting on October 24, 2022.

## Materials and methods

We used the National Inpatient Sample (NIS) database, which is part of the Healthcare Cost & Utilization Project. The national cohort database comprises data on over seven million hospital stays within the United States and reflects approximately 20% of discharges in the United States.

We extracted inpatient data for the years 2016 through 2019 (the latest year available in the database at the time of data extraction) from patients with a primary discharge diagnosis of cirrhosis as defined by ICD code B9681. Patients with concomitant diagnosis of *H. pylori* infection were extracted as defined by ICD code K70. Baseline characteristics were compared between *H. pylori*-infected and non-*H. pylori*-infected patients, including demographic factors (age, gender, race), comorbid conditions, Elixhauser Comorbidity Index, median household income, primary payer, hospital characteristics, disposition, and admission type. Primary outcomes were inpatient mortality, length of stay, and cost of care.

The secondary endpoints comprised complications related to cirrhosis during hospitalization, such as gastrointestinal bleeding, hepatic encephalopathy, hepatorenal syndrome, and spontaneous bacterial peritonitis, defined using appropriate ICD codes. Adjustment for potential confounding variables, including age, race, female gender, hypertension, diabetes, hyperlipidemia, heart failure, chronic kidney disease (CKD) stage 3 or greater, alcohol use disorder, and coronary artery disease, was conducted through multivariable logistic regression analysis.

Patients were categorized into two mutually exclusive groups: one with *H. pylori* and the other without. Demographic characteristics and in-hospital outcomes were analyzed for both groups. Mean values with 95% confidence intervals were used for continuous variables, while categorical variables were expressed as percentages of the total population. We utilized Healthcare Cost and Utilization Project (HCUP) weights and STATA's svy functions to estimate the national hospitalized US population from the observed hospitalization-level data. Differences between groups were assessed using t-tests and Chi-square tests for continuous and categorical variables, respectively, with a significance threshold of p<0.05.

The Elixhauser Comorbidity Index was computed using SAS version 9.4. Univariate logistic analysis was employed to examine the association between *H. pylori* and various outcomes, such as overall complication rates, in-hospital mortality, encephalopathy, gastrointestinal bleeding, spontaneous bacterial peritonitis, and hepatorenal syndrome, in comparison to patients without *H. pylori*. Subsequently, a multivariate logistic regression analysis was conducted for the same outcomes, adjusting for baseline factors that were significantly different (p<0.1) in baseline characteristics according to univariate analysis. The results of regression analyses were presented as odds ratios (OR) with corresponding confidence intervals (CI) and p-values. All statistical calculations were performed using STATA Version 14.2.

## Results

Baseline characteristics

From the years 2016 to 2019, 416,410 patients had a primary discharge diagnosis of cirrhosis (Table 1). Of those patients, 990 (0.2%) had a secondary diagnosis of *H. pylori* infection. Cirrhosis patients with *H. pylori* were younger on average (mean age 54.25 vs. 57.18 years, p=0.01) and more likely to be within ages 18-49 (33.84% vs 24.71%, P=0.01). *H. pylori*-infected patients were also more likely to identify as male (70.71% vs 63.11%, P<0.028), Hispanic race (36.4% vs. 18.6%, p< 0.1), and of Black race (20.2% vs. 8.1%, p< 0.1). *H. pylori* patients were additionally more likely in the bottom quartile of median household income (48.17% vs. 34.66%, p< 0.01). The Elixhauser Comorbidity Index for mortality was similar in *H. pylori* and non-*H. pylori* patients (-1.77 vs -1.86, P=0.809).

**Table 1:** Baseline characteristics of the study population To estimate the national hospitalized US population from the observed hospitalization-level data, we utilized weights provided by HCUP along with the svy set and svy functions in STATA. We employed t-tests and Chi-square tests to examine differences between groups concerning both continuous and categorical variables. Statistical significance was determined by p-values less than 0.05. ^a^Comorbidities were coded using the appropriate ICD-10 in the secondary diagnosis field. ^b^Quartile classification of estimated median household income of residents in the patient's zip code (demographic data obtained from Claritas) was performed, with values 1 to 4 indicating the poorest to wealthiest populations. These are estimates, regularly updated, and subject to potential year-to-year variations. More details can be found at https://www.hcup-us.ahrq.gov/db/vars/zipinc_qrtl/nisnote.jsp ^c^Federal insurance was defined if the primary payer was either Medicare or Medicaid. All other categories were categorized as private insurance. Additional information is available at https://www.hcup-us.ahrq.gov/db/vars/pay1/nisnote.jsp ^d^Bed size cutoffs were categorized into small, medium, and large based on hospital beds, with specifications specific to the hospital's location and teaching status. Further details can be found at https://www.hcup-us.ahrq.gov/db/vars/hosp_bedsize/nisnote.jsp ^e^A teaching hospital was identified if it was a member of the Council of Teaching Hospitals (COTH) had an AMA-approved residency program, or had a ratio of full-time interns and residents to beds of 0.25 or higher. Additional information can be found at https://www.hcup-us.ahrq.gov/db/vars/hosp_locteach/nisnote.jsp ^f^Elixhauser Comorbidity Software Refined for ICD-10-CM 2021, Agency for Healthcare Research and Quality R, Rockville, MD. www.hcup-us.ahrq.gov/toolssoftware/comorbidityicd10/comorbidityicd10.jsp

Baseline Characteristics	Overall (%) (N = 416,410)	*Helicobacter pylori* Absent (%) (N = 415,420)	*Helicobacter pylori* Present (%) (N = 990)	P-value
Cirrhosis				
Mean Age, (Mean ± Standard error) (Years)	57.17 (57.07 - 57.27)	57.18 (57.08 - 57.28)	54.25 (52.55 - 55.95)	0.001
Age (years)				0.01
• 18-49	24.73	24.71	33.84	0.01
• 50-64	49.61	49.61	47.47	0.01
• 65-74	17.83	17.84	12.12	0.01
•>= 75	7.84	7.84	6.57	0.01
Gender				0.028
• Male	63.13	63.11	70.71	0.028
• Female	36.87	36.89	29.29	0.028
Race				<0.001
• White	64.01	64.10	26.26	<0.001
• Black	8.13	8.10	20.20	<0.001
• Hispanic	18.62	18.58	36.36	<0.001
• Asian or Pacific Islander	1.72	1.71	5.56	<0.001
• Native American	1.50	1.50	2.53	<0.001
• Other	6.02	6.02	9.09	<0.001
Comorbidity^a^				
• Obesity	14.29	14.30	11.62	0.275
• Body mass index (BMI) < 20	2.71	2.71	3.54	0.578
• Hypertension	49.70	49.71	42.42	0.042
• Diabetes	30.10	30.12	24.75	0.108
• Hypercholesterolemia	16.33	16.35	8.08	0.002
• Heart failure	11.95	11.96	7.58	0.059
• Valvular disease	0.74	0.74	0.51	0.701
• Chronic kidney disease (CKD) stage 3 or more	12.92	12.94	6.06	0.004
• Alcohol use disorder	48.56	48.53	61.11	<0.001
• Peripheral vascular disease	3.72	3.73	1.52	0.101
• Chronic obstructive pulmonary disease (COPD)	12.94	12.94	10.61	0.327
• Stroke	0.58	0.59	0.00	0.295
• Coronary artery disease (CAD)	12.19	12.21	7.07	0.028
• Cardiomyopathy	2.28	2.28	2.53	0.818
• Coagulopathy	0.86	0.86	0.00	0.219
• Bleeding disorder	42.48	42.48	41.92	0.873
• Smoking	22.28	22.27	25.25	0.325
• Cancer	7.70	7.70	6.06	0.389
• Autoimmune disease	2.06	2.06	1.01	0.298
Median Household Income Category for Patient's Zip Code^b^				0.0002
• 0-25 percentile	34.69	34.66	48.17	0.0002
• 26-50 percentile	26.73	26.73	24.61	0.0002
• 51-75 percentile	22.64	22.64	19.90	0.0002
• 76-100 Percentile	15.94	15.96	7.33	0.0002
Primary Payer^c^				0.002
• Federal insurance	65.68	65.68	65.68	0.002
• Private insurance	21.86	21.88	12.12	0.002
• Other	3.22	3.22	5.56	0.002
• Uninsured	9.06	9.05	15.66	0.002
• Missing	0.17	0.17	0.00	0.002
Hospital Characteristics				
Hospital Region				0.013
• Northeast	16.76	16.76	17.17	0.013
• Midwest	19.27	19.27	19.19	0.013
• South	43.00	43.02	33.84	0.013
• West	20.97	20.95	29.80	0.013
Hospital Bed Size^d^				0.025
• Small	17.11	17.11	13.64	0.025
• Medium	28.33	28.35	22.22	0.025
• Large	54.56	54.54	64.14	0.025
Hospital Teaching Status^e^				<0.001
• Non-teaching	26.42	26.45	15.15	<0.001
• Teaching	73.58	73.55	84.85	<0.001
Hospital Location				0.066
• Rural	5.51	5.52	2.53	0.066
• Urban	94.49	94.48	97.47	0.066
Disposition				<0.001
• Home	63.25	63.21	80.30	<0.001
• Facility/others	4.43	4.44	0.51	<0.001
• Died	32.32	32.35	19.19	<0.001
Admission Type				0.446
• Non-Elective	94.70	94.70	97.47	0.446
• Elective	5.14	5.14	2.53	0.446
• Missing	0.16	0.16	0.00	0.446
Elixhauser Comorbidity Index for Mortality (Mean ± 95% Conf. Interval)^f^	-1.86 (-1.91 to -1.81)	-1.86 (-1.91 to -1.81)	-1.77 (-2.51 to -1.03)	0.809

Primary outcomes

*H. pylori*-exposed patients had lower in-hospital mortality (0.51% vs 4.44%, p=0.007) (Table 2). The mean length of stay, however, was higher in the *H. pylori* group (6.97 days vs 5.75, p=0.002). The overall cost of care was similar between the *H. pylori* exposed and non-exposed groups (mean USD18,106.18 vs $16,543.49, P=0.160).

**Table 2:** Outcomes during hospitalization The results of the statistical analysis for outcomes during hospitalization are presented here. With the weights provided by HCUP and svyset in STATA, we generated a national estimate of the hospitalized US population from our observed data collection. P-values of less than 0.05 were considered statistically significant.

Outcome	Overall (%) (N= 416,410)	Helicobacter pylori Absent (%) (N= 415,420)	Helicobacter pylori Present (%) (N= 990)	P-value
Cirrhosis				
In-hospital mortality	4.43	4.44	0.51	0.007
Length of stay (Mean ± Standard error) (Days)	5.76 (5.68 - 5.83)	5.75 (5.68 - 5.83)	6.97 (6.21 - 7.72)	0.002
Cost of care (Mean ± Standard error) (USD)	16547.22 (16031.09 - 17063.36)	16543.49 (16026.53 - 17060.45)	18106.18 (15947.94 - 20264.41)	0.160

Complications of cirrhosis

**Figure 1:**
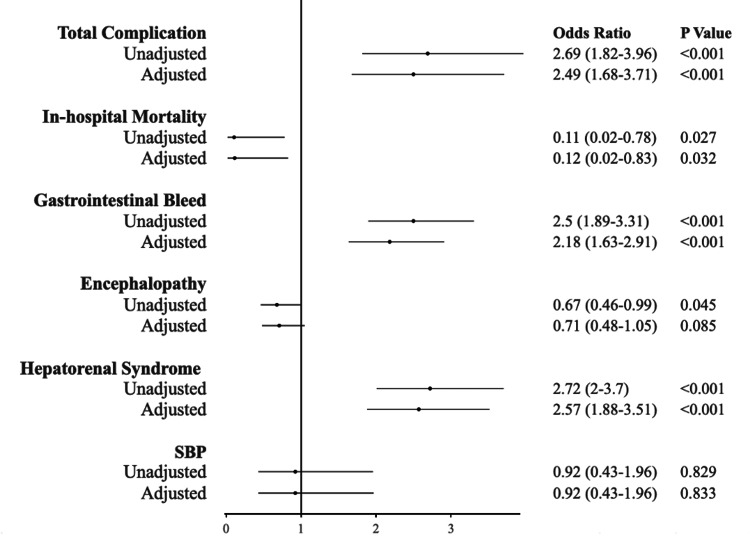
Adjusted outcomes for potential confounders using multivariable logistic regression analysis Odds of complication in cirrhosis patients with *H. pylori* infection compared to those without infection SBP: spontaneous bacterial peritonitis

Patients exposed to *H. pylori* exhibited a higher overall incidence of complications related to cirrhosis (84.8% vs. 67.5%, p < 0.001), gastrointestinal bleeding (48.4% vs. 27.3%, p < 0.001), and hepatorenal syndrome (70.7% vs. 46.9%, p < 0.001), and these differences persisted even after adjusting for multiple variables in the analysis (refer to Table 3). Initially, rates of hepatic encephalopathy were higher in patients not exposed to *H. pylori* (21.5% vs. 15.6%, p <0.04), but this was rectified after accounting for potential confounding factors in the multivariate analysis, including age, race, sex, hypertension, diabetes mellitus, hyperlipidemia, heart failure, chronic kidney disease stage 3 or greater, alcohol use disorder, and coronary artery disease (Figure 1).

**Table 3:** Complications during hospitalization For this data set, we used the same weights provided by HCUP and svy functions in STATA to generate a national estimate of the population based on what we observed from our data collection. The differences were tested for variables using the t-test and Chi-square tests, and a p-value of less than 0.05 was considered statistically significant. ^a^Complications were coded using appropriate ICD-10 in the secondary diagnosis field

Complications^a^	Overall (%) (N= 416,410)	Helicobacter pylori Absent (%) (N= 415,420)	Helicobacter pylori Present (%) (N= 990)	P-value
Cirrhosis				
Gastrointestinal bleed	27.39	27.34	48.48	<0.001
Hepatic encephalopathy	21.56	21.57	15.66	0.040
Hepatorenal syndrome	47.04	46.99	70.71	<0.001
Spontaneous bacterial peritonitis	3.83	3.83	3.54	0.826
Death	4.43	4.44	0.51	0.007
Overall complication	67.63	67.59	84.85	<0.001

## Discussion

*H. pylori* is a significant cause of morbidity and mortality, given its high prevalence worldwide. It was first discovered and identified as a cause of peptic ulcer disease in 1982 by Dr. Warren and Professor Marshall [6]. Its ability to colonize the stomach and cause gastrointestinal pathology is derived through four key steps: urease production, which confers acid neutralization within the stomach, flagella-mediated motility, cellular attachment through adhesins, and toxin production (e.g., CagA, VacA) causing tissue damage. CagA is also considered a major oncogenic factor in the development of gastric cancer. *H. pylori* has been classified as a group I carcinogen by the WHO since 1994 and is the etiologic factor in approximately 75% of worldwide gastric cancers and 5.5% of total malignancies [6-7].

*H. pylori* may be an independent contributor to liver damage through oxidative stress and hepatocyte injury [8]. This may fuel the development of decompensating events in cirrhosis. In particular, the association between hepatic encephalopathy and *H. pylori* has been of interest, given its ability to form ammonia. However, *H. pylori* eradication has not been consistently found to decrease ammonia levels and improve hepatic encephalopathy [9]. Other studies have shown an increased incidence of esophageal varices, gastric variceal bleeding, portal vein thrombosis, portal hypertensive gastropathy, and the development of hepatocellular carcinoma [10-13]. The findings of our multivariable analysis were significant for higher overall rates of gastrointestinal bleeding and hepatorenal syndrome in *H. pylori*-exposed patients.

There are multiple mechanisms by which *H. pylori* disturbs normal tissue. For example, it can release toxins directly into the cytoplasm or increase IL-8 and hydrogen peroxide in epithelial cells. It has been hypothesized that *H. pylori* can cause an overall state of severe endothelial dysfunction, including the portal venous system. This would lead to increased stiffness of the portal vein, increased portal pressure, and contribute to the formation of portal hypertension [14]. Portal hypertensive gastropathy results from intramucosal hemorrhage caused by portal hypertension and is characterized endoscopically by a mosaic-like pattern that may clinically manifest as acute or chronic gastrointestinal bleeding. This chronic inflammatory state formed by *H. pylori* can directly and indirectly further impair hepatocytes, exacerbating portal hypertensive gastropathy and increasing the risk of inflammatory lesions within the stomach in cirrhotic patients [10]. In addition, the overproduction of these pro-inflammatory cytokines can worsen portal hypertension and negatively affect varices. The current literature supports a significant association between *H. pylori* infection and gastrointestinal bleeding in both non-variceal and variceal bleeding forms [11]. A study by Devrajani et al., focusing on *H. pylori* infection in cirrhotic patients with upper gastrointestinal bleeding, demonstrated a 56% infection rate in these patients [15]. Another prospective study conducted by Elsebaey et al. demonstrated a significantly higher prevalence of *H. pylori* infection in cirrhotic patients with variceal bleeding than those without. It has been proposed that hypergastrinemia induces a hyperacidic state that is detrimental to the gastric mucosa causing erosions, ulcerations, and eventual variceal rupture and bleeding in cirrhotic patients [11]. The results of the current study showed higher rates of complications from gastrointestinal bleeding in *H. pylori*-infected patients compared with those without infection, which is consistent with the current literature.

In the present study, the rate of hepatic encephalopathy was initially higher in non-infected patients; however, this was corrected after multivariable analysis. Hepatic encephalopathy is a frequent complication of cirrhosis, with its pathogenesis thought to be driven by hyperammonemia. Therefore, many therapeutic approaches aim to target ammonia, such as targeting glutamine metabolism in the small bowel or bacterial ammonia production in the large bowel. However, the stomach can be another source of ammonia if *H. pylori* is present. As noted previously, *H. pylori* has the ability to form ammonia due to its urease activity, causing serum ammonia levels to increase at an even higher rate in cirrhotic patients and potentially accelerate hepatic encephalopathy [16]. Chen et al. conducted a prospective study that found hepatic encephalopathy was more frequently observed in cirrhotic patients infected with *H. pylori*. It also noted reduced levels of blood ammonia as well as significantly lower rates of hepatic encephalopathy after *H. pylori* eradication. On the other hand, other published literature on this association suggests that the ammonia levels produced by *H. pylori* are likely clinically insignificant in most cirrhotic patients. Despite multiple observational and interventional studies, it is still unclear whether clinicians should look for and treat *H. pylori* infection in all cirrhotic patients with hepatic encephalopathy [16-17].

Through multivariable analysis, this study found higher rates of hepatorenal syndrome in cirrhotic patients with *H. pylori*. *H. pylori* infection induces the release of multiple inflammatory cytokines and vascular active substances, such as C-reactive protein (CRP), tumor necrosis factor-alpha (TNF-α), interleukin 1 (IL-1), interleukin 6 (IL-6), interleukin 8 (IL-8), heat shock protein (HSP) causing local and systemic reactions [18]. Through risk factors of renal endovascular damage, proteinuria, and mesangial proliferation, *H. pylori*-exposed patients have accelerated loss of kidney function [18]. The development of renal failure in cirrhotic patients is known as hepatorenal syndrome, characterized by marked renal vasoconstriction with peripheral arterial vasodilation. A precipitating event, such as gastrointestinal bleeding, can place cirrhotic patients at a higher risk of developing hepatorenal syndrome [19]. As presented earlier, *H. pylori*-infected cirrhotic patients have been shown to have higher rates of gastrointestinal bleeding versus those without infection. In addition, a single-center observational study researching *H. pylori* infection as an initiating factor of complications in cirrhotic patients found an increased incidence of hepatorenal syndrome in *H. pylori*-positive versus negative patients [13]. The literature on the association between *H. pylori* and hepatorenal syndrome is limited. More studies will need to be conducted to understand the pathophysiology and determine whether this truly represents the general population.

## Conclusions

This retrospective analysis has inherent limitations, and it is essential to acknowledge them. While the patients identified in the study were diagnosed with both *H. pylori* and cirrhosis upon discharge, it cannot be definitively concluded that *H. pylori* directly caused the complications associated with cirrhosis. Recognizing this uncertainty, further research is needed to better understand the connections between cirrhosis and complications related to *H. pylori*. Investigating the origins of cirrhosis and its relationship with *H. pylori* could offer valuable insights into whether the presence of *H. pylori* is a causative factor or merely a correlated aspect in patients with cirrhosis. Additionally, a prospective study focusing on eradicative therapy for *H. pylori* might prove beneficial in specific cases of cirrhosis, helping to determine whether such treatment can reduce the incidence of complications associated with cirrhosis.
